# Comparative Approach to the Temporo-Spatial Organization of the Tumor Microenvironment

**DOI:** 10.3389/fonc.2019.01185

**Published:** 2019-11-07

**Authors:** Kendall L. Langsten, Jong Hyuk Kim, Aaron L. Sarver, Mark Dewhirst, Jaime F. Modiano

**Affiliations:** ^1^Department of Veterinary Population Medicine, University of Minnesota, St. Paul, MN, United States; ^2^Animal Cancer Care and Research Program, University of Minnesota, St. Paul, MN, United States; ^3^Department of Veterinary Clinical Sciences, College of Veterinary Medicine, University of Minnesota, St. Paul, MN, United States; ^4^Masonic Cancer Center, University of Minnesota, Minneapolis, MN, United States; ^5^Institute for Health Informatics, University of Minnesota, Minneapolis, MN, United States; ^6^Radiation Oncology Department, Duke University Medical School, Durham, NC, United States; ^7^Department of Laboratory Medicine and Pathology, University of Minnesota Medical School, Minneapolis, MN, United States; ^8^Center for Immunology, University of Minnesota, Minneapolis, MN, United States; ^9^Stem Cell Institute, University of Minnesota, Minneapolis, MN, United States; ^10^Institute for Engineering in Medicine, University of Minnesota, Minneapolis, MN, United States

**Keywords:** tumor microenvironment, temporo-spatial organization, dog, canine, human

## Abstract

The complex ecosystem in which tumor cells reside and interact, termed the tumor microenvironment (TME), encompasses all cells and components associated with a neoplasm that are not transformed cells. Interactions between tumor cells and the TME are complex and fluid, with each facet coercing the other, largely, into promoting tumor progression. While the TME in humans is relatively well-described, a compilation and comparison of the TME in our canine counterparts has not yet been described. As is the case in humans, dog tumors exhibit greater heterogeneity than what is appreciated in laboratory animal models, although the current level of knowledge on similarities and differences in the TME between dogs and humans, and the practical implications of that information, require further investigation. This review summarizes some of the complexities of the human and mouse TME and interjects with what is known in the dog, relaying the information in the context of the temporo-spatial organization of the TME. To the authors' knowledge, the development of the TME over space and time has not been widely discussed, and a comprehensive review of the canine TME has not been done. The specific topics covered in this review include cellular invasion and interactions within the TME, metabolic derangements in the TME and vascular invasion, and the involvement of the TME in tumor spread and metastasis.

## Introduction

**Figure 1:**
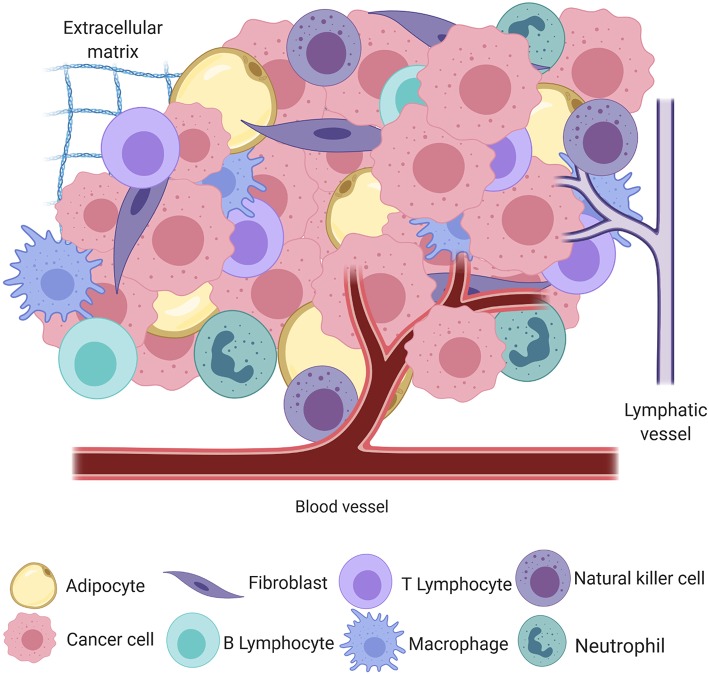
A simplified schematic of the cellular and structural component of the tumor microenvironment, including adipocytes, fibroblasts, B and T lymphocytes, macrophages, natural killer cells, neutrophils, blood and lymphatic vessels, and the extracellular matrix, all intermingled with transformed cancer cells (created with Biorender.com).

Cancer, the uncontrolled proliferation of cells, is a significant cause of morbidity and mortality in humans and their canine companions worldwide (1, 2). The process of neoplastic transformation is similar amongst species and can most easily be conceptualized in the three steps of initiation, promotion, and progression toward malignancy (3), although it is now apparent that these steps are neither sequential nor obligate. In the seminal work by Hanahan and Weinberg (4), tumors were introduced as complex heterotypic tissues where a non-transformed milieu influences the progression of transformed cells with which it co-exists in the same space and time. This milieu, the tumor microenvironment (TME), may be thought of as the ecosystem or community within which neoplastic cells survive and reside. The genomic landscape of the malignant cells and the composition and behavior of the TME are shaped by intense selection that can be described as prototypical Darwinian evolution in a microscopic scale. All non-transformed cells that interact with tumor cells, including inflammatory cells, endothelium, adipocytes, and fibroblasts, among others, as well as the non-cellular components, including structural scaffold surrounding the cells and the soluble factors secreted by the tumor and non-tumor components, compose the TME [Figure 1; (5)]. In a non-neoplastic environment, these components have a vast range of functions, including forming the interstitium that creates a scaffold for parenchyma to organize, sequestering growth factors, supplying nutrients, draining waste from tissue, and creating a competent immune system to protect the body against invaders.

**Figure 2:**
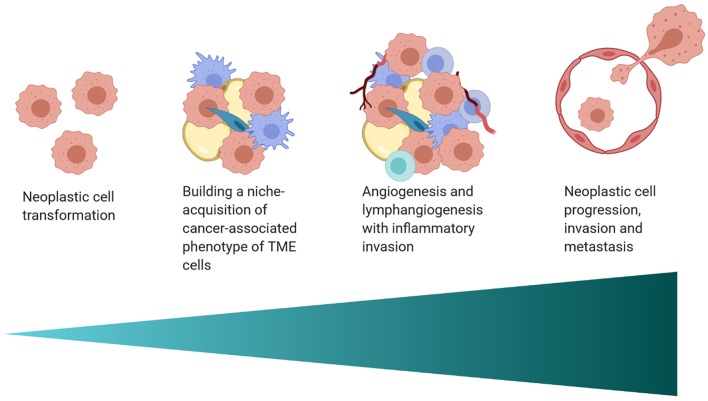
A schematic of the proposed temporo-spatial organization of the TME. Cells must first undergo neoplastic transformation, allowing for the creation of a permissive micro-ecological niche. Neighboring cells, including adipocytes, fibroblasts, and macrophages, among others, can adopt a cancer-associated phenotype, with complex, pro-tumorigenic effects. Hypoxia induced by cell proliferation and metabolic changes encourage lymph and blood vessel invasion, increasing infiltration by inflammatory cells. Furthermore, angiogenesis and lymphangiogenesis create increased opportunity for neoplastic cell spread and metastasis (created with Biorender.com).

The interplay between the TME and tumor cells is paramount in the progression and response to neoplastic growth. While our understanding of the TME in dogs is rudimentary, there are the similarities in tumor heterogeneity between dogs and humans (Table 1), which are often not appreciated in laboratory animal models. Although the current level of knowledge on similarities and differences in the TME between dogs and humans, and the practical implications of that information require further investigation. This review provides an overview of the complexity observed in the human and mouse TME, interjects known similarities and differences in the dog, and relays them in the context of the temporo-spatial organization of the TME. In short, the proposed temporo-spatial organization of the TME involves neoplastic tells transforming, the transformation of adjacent TME into a cancer associated phenotype, and vascular invasion, potentially culminating in tumor cell spread and metastasis (Figure 2). To the authors' knowledge, this approach to the organization and conceptualization of the TME, as well as a review of the TME in the dog, have not been described before. Discussions include cellular invasion and interactions within the TME, metabolic derangements in the TME and vascular invasion, and the involvement of the TME in tumor spread and metastasis.

**Table 1:** Comparative features of the TME between dogs and humans.

**Components of the TME**	**Dog**	**Human**
Adipocytes	Produce aromatase cytochrome P450, estrogen, and progesterone which stimulates tumor development	
Adipose-derived mesenchymal stem cells	Suppress T cells through TGFβ and adenosine pathways	Suppress T cells through indoleamine 2,3-dioxygenase (IDO) pathway
Fibroblasts	Unknown	Matrix is capable of inhibiting tumor cell spread
Cancer-associated fibroblasts	Modulate gene expression of cancer cells	
Soluble factors	IL-8 receptors are upregulated on cancer cells, leads to increase in angiogenesis and inflammation	
	Elevated Cox-2 levels in certain tumor types; Cox-inhibitors utilized for anti-tumor effects	
Lymphatics	Density of lymphatic vessel is correlated with tumor growth and metastasis	
Immune cells	Osteosarcoma can be separated into “hot” (active) and “cold” (barren) tumors, in regards to inflammatory response	
	Increased presence of immune transcripts in osteosarcoma is not prognostically significant	Increased presence of immune transcripts in osteosarcoma is associated with better prognosis

## Cellular Invasion and Interactions Within the TME

While the definition of “cancer stem cells” (CSCs; also called tumor-initiating cells or tumor-propagating cells) is mostly semantic, the importance of cells that retain or acquire stem-like features in the tumor cannot be underestimated. Whether they be few, as in traditional hierarchically-organized tumors, or many, as in stochastically organized tumors, these cells contribute to remodeling the TME (6).

Recent work characterized genome-wide gene expression signatures in canine tumor models (hemangiosarcoma, osteosarcoma, and glioblastoma) that were grouped according to their hierarchical organization (7). Cell lines derived from these three tumor types were cultured under non-adherent low serum conditions that promote sphere formation and enrich CSCs. The steady state gene expression associated with CSC maintenance in tumors with high sphere-forming efficiency (i.e., hierarchically organized with relatively few CSCs) showed metabolic skewing toward fatty acid synthesis and secretion of immunosuppressive cytokines. On the other hand, tumors with low sphere-forming efficiency (i.e., stochastically organized with many or most cells having CSC potential) showed metabolic skewing toward fatty acid oxidation and potential immunoevasion through upregulation of CD40. In the incipient tumor, CSCs create reactive microenvironments that support tumor growth (8), importantly, by producing cytokines that leverage the innate properties of resident macrophages to remodel the microenvironment. CSCs also interact bidirectionally with myeloid cells, which can remain in an incompletely differentiated state and become myeloid-derived suppressor cells (MDSCs, a highly heterogeneous population of cells that contributes to cancer stemness as well as to the functional immunosuppressive barrier (9, 10).

Intriguingly, the CSC condition appears to be at least partly under extrinsic control. Depletion of CSCs in cultured canine and human cell lines leads to reprogramming of differentiated cells to become CSCs, maintaining the population in a steady state (11). The signals that regulate this process are poorly understood, but they might involve dysregulated expression of Snail family transcription factors Snail (SNAI1), Slug (SNAI2), as well as TWIST1 and Zeb1 (12–14), perhaps through epigenetic modification of their respective promoters (15). These responses are tightly regulated by environmental cues. For example, expression of SNAI2 and its targets, CDH1, VIM, and JUP in hemangiosarcoma cells showed a biphasic response to interkeukin-8 (IL-8), with small amounts of IL-8 favoring self-renewal and abundant IL-8 favoring expansion of bulk (differentiated) tumor cells (8, 16).

The role of adipocytes in the TME has received more attention as evidence mounts for a link between obesity and cancer risk in dogs and humans (17, 18). Adipocytes adjacent to tumor cells, known as cancer-associated adipocytes (CAAs), are recruited to be actively involved in tumor initiation, promotion, and progression. The mechanism of CAA development is unclear, but likely involves a bidirectional communication stream that includes adipokines and extracellular vesicles, among other factors. Adipokines, metabolically active substances secreted by adipocytes to create a permissive TME, include substances such as leptin, tumor necrosis factor-α (TNFα), C-C Motif Chemokine Ligand 2 (CCL2), and adiponectin. A concise review of adipokines in domestic animals was recently published (19).

Adipocytes promote neoplastic development through a variety of mechanisms, including supporting angiogenesis (described later in this review), manipulating tumor cell metabolism, and encouraging a pro-inflammatory state, leading to the recruitment of macrophages. Adipocytes play an important role in reprogramming tumor cell metabolism. For example, ovarian cancer cells co-cultured with abdominal adipose cells were shown to coerce neighboring adipocytes into supplying free fatty acids, thereby providing substrates for sustained tumor cell replication (20). The role of adipocytes in promoting chronic inflammation has been the subject of numerous studies. Adipocytes produce pro-inflammatory adipokines and cytokines [including CCL2, interleukin-6 (IL-6), and TNFα], which increase inflammation and metastatic risk, supporting tumor survival (20, 21). In the case of mammary and breast carcinomas in dogs and humans, respectively, adipocyte-derived aromatase cytochrome P450, estrogen, and progesterone have been reported to stimulate tumor development and enhance invasive potential [Table 1; (22, 23)]. Finally, extracellular remodeling in tumors, including increased collagen deposition by adipocytes, can lead to adipocyte apoptosis and necrosis. Macrophages are then recruited into the tumor due to the release of pro-inflammatory damage-associated molecular patterns (DAMPs) from the dying adipocytes (17).

Recently, attention has been given to the influence of adipose-derived mesenchymal stem cells (ad-MSCs) in tumor progression. The secretome of ad-MSCs is incompletely understood but is thought to have overarching immunomodulatory and pro-angiogenic properties (24). The immunomodulatory properties of these cells are dependent on the inflammatory milieu in which the cells reside. Some of the anti-inflammatory properties of human and dog MSCs seem to differ mechanistically. Ad-MSC dependent T cell suppression in humans is through the indoleamine 2,3-dioxygenase (IDO) pathway, resulting in decreased T cell function through tryptophan depletion (25). Alternatively, in dogs ad-MSCs most likely decrease T cell activity through TGFβ and adenosine pathways [Table 1; (26)]. While a solid body of knowledge about the influence of adipocytes and ad-MSCs in human tumor growth and progression has been developed in recent years, the influence of these cells on the TME in dogs remains to be elucidated.

In a non-cancer associated microenvironment, fibroblasts play a major role in producing components of the extracellular matrix (ECM) including fibrillar collagen, elastin, laminin, fibronectin, and glycosaminoglycans (27). Fibroblasts are critical in wound healing, inflammatory reactions, fibrosis, promoting angiogenesis, and cancer progression. Tumors are often conceptualized as a “wound that will not heal” with abundant collagen deposition (28). *In vitro* studies using cell lines from various species, although to the authors' knowledge not from dogs, have demonstrated that normal, non-cancer associated fibroblasts and the matrix they produce are capable of inhibiting the spread of tumor cells, a phenomenon termed neighbor suppression (29–31). Since neighbor suppression was first recognized by Stoker et al. (29), many theories have developed around the molecular mechanisms influencing this finding, including heterologous communication between transformed and non-transformed cells through junctional complexes and through soluble factors within the ECM (32, 33). Neighbor suppression has not yet been recognized in canine tumors (Table 1).

Cancer-associated fibroblasts (CAFs) are corrupted by the neoplastic cells in their proximity and have drastically different functions than their non-transformed counterparts. The origin of CAFs is not entirely clear; many theories on their origin claim CAFs originate from resident mesodermal precursors (34–38). An influential paper by Erez et al. (39) demonstrated that the transcription factor NFκB induces the CAF phenotype through upregulation of pro-inflammatory genes. These findings suggest a necessity for innate immune involvement in the education of CAFs. Furthermore, epigenetic changes also play a role in the development of CAFs. Albrengues et al. (36) demonstrated that CAFs have constitutively activated JAK1/STAT3 signaling pathways secondary to epigenetic changes. Histone acetylation of STAT3 in CAFs by leukemia inhibitory factor (LIF) caused subsequent activation of DNMT3b (a DNA methyltransferase). This in turn led to decreased SHP-1 expression with subsequent sustained activation of JAK1. Interestingly, inhibition of DNMTs caused CAFs to convert to a non-cancer associated fibroblast phenotype (36). CAFs have diverse phenotypes without unique markers, although phenotypic similarities to myofibroblasts, including reduced caveolin-1 (CAV-1) expression and increased expression of α-SMA, vimentin, fibroblast-activating protein, and MCT-4 (40, 41) have been described. Additionally, CAFs have been shown to increase tumor cell growth, motility, and local invasion through ECM remodeling and cytokine release (37, 42, 43). In both humans and dogs, CAFs modulate gene expression of cancer cells (44, 45). However, it is difficult to compare their transcriptional programs across species, as experimental protocols and genes of interest differ between published studies. Functionally, CAFs differ from normal fibroblasts in the products and quantities of enzymes that they produce. For example, in both canine mammary carcinoma and human breast carcinoma CAFs exhibit increased aromatase activity, which is associated with hormone-driven tumor progression (46, 47).

Mesenchymal stem cells (MSCs), also known as undifferentiated fibroblasts or mesenchymal stromal cells, are another important component of the TME. These cells are phenotypically plastic cells that originate from the mesoderm (48). MSCs home from bone marrow, spleen and other locations to sites of injury and inflammation, including tumors (49). The role of MSCs in the TME are numerous; one of the better-studied functions is their influence in changing the immune landscape (for more information, see the section on metabolism, vascular invasion, and immune cells within the TME).

Tumor-associated ECM is markedly different from ECM in a non-pathologic milieu. As an active driver of tumor progression, tumor-associated ECM is reorganized, directing tumor cell migration and promoting local invasion along collagen fibers (50, 51). Furthermore, tumor-associated ECM is associated with increased pro-inflammatory cytokines, promotes angiogenesis, and factors that increase fibroblast proliferation (52). As all components of the TME are simultaneously interacting with one another and tumor cells, it stands to reason that by encouraging inflammation, tumor-associated ECM likely contributes to the production of CAFs. Collagen is one of the most abundant components of the ECM and is known to exhibit tumor-associated collagen signatures. Differences in collagen density, width, length, and straightness, as well as reorganization of the boundary between tumor and stroma, are some of the collagen signatures appreciated (53, 54). In dogs and humans, collagen signatures are important prognostic indicators in mammary and breast carcinoma (53, 54). For example, in a study analyzing characteristics of mammary carcinoma in dogs, tumor-associated ECM had upregulated collagen1α1, α-SMA, fibroblast activation protein (FAP), platelet-derived growth factor (PDGF)-β, and paradoxically, CAV-1 (55).

Interactions between tumor cells, stromal cells, and the ECM are heterogeneous and tumor-specific. However, fragmentation of hyaluronic acid, which is pro-inflammatory, and deposition of tenascin-C seem to occur in most tumor types (56–58). The hyaluronic acid receptor, CD44, is expressed by most tumor and stromal cells, but the highest levels are seen in CSCs (6). In addition to HA, the ECM is composed of collagens, elastins, laminins, fibrinogen, and tissue-specific proteoglycans. The stoichiometry and topology of these components regulates adhesion (for example, by interaction with cell surface integrins) and stiffness of the ECM. Tumor cells and inflammatory cells secrete proteases that degrade the ECM, and proteins and proteoglycans to remodel it. The interactions of the ECM with integrins, mechanoreceptors, and signaling proteins that activate contractility, such as focal adhesion kinase, modulate cellular motility, proliferation and survival (59–61). The interactions are bidirectional, as the cytoskeleton “pushes back” into the ECM, maintaining integrins and focal adhesions in a state of isometric tension. Increased tension also activates the small G protein Rho and its target Rho-associated kinase (ROCK), which controls myosin light chain phosphorylation. The ECM in most tumors is several orders of magnitude stiffer than their normal tissue counterparts, making it permissive for cell migration and ultimately, metastasis (59). There are myriad studies documenting the importance of ROCKs in tumor progression, but a noteworthy study showed that ROCK inhibitors were able to push chemoresistant mouse osteosarcoma cells away from a malignant phenotype and into terminal adipocyte differentiation (62). Perhaps more interestingly, cells that escaped terminal differentiation in the presence of ROCK inhibitors regained sensitivity to chemotherapy and could be eliminated by treatment with doxorubicin (62).

The extensive heterogeneity and adaptation of the tumor niche is partly dependent on intercellular communication. Malignant cells co-opt developmental programs of intercellular communication to create and maintain a niche with unique properties that promote growth and survival (6). Intercellular communication involves a multitude of interactions mediated by cell-to-cell contacts and soluble mediators. Cell-to-cell contacts include adhesion molecules, stable ligand-receptor interactions, and promiscuous, transient to stable interactions between cell surface proteins, glycans, and lipids (63, 64). Emerging evidence also suggests that cells can communicate in the local environment by exchanging genetic and biochemical mediators through tunneling nanotubes (65, 66). Soluble mediators of communication include hormones, cytokines, chemokines, lipids, and microvesicles (67–70). Cells also interact with their external environment through pressure receptors and by molding the ECM (59, 71–73).

Soluble mediators of communication have been relatively well-described in humans, although there is little information available as to the impact of the stroma and soluble factors in dogs. Kim et al. (8) showed that IL-8, a cytokine produced by fibroblasts, neoplastic cells, and other cell types, supports tumor progression by modulating the TME in canine hemangiosarcoma into a more “reactive” state; increasing the propensity toward inflammation, fibrosis, and coagulation. Intriguingly, IL-8 blockade reduced tumor cell survival and engraftment in a xenograft model of canine hemangiosarcoma, indicating this cytokine may be necessary to establish the initial niche for this disease (8). Similar findings have been reported in humans, with tumor cells of various tumor types upregulating IL-8 production and IL-8 receptors on cancer cells as well as other cells types with increases in angiogenesis and inflammation within those tissues [Table 1; (74, 75)]. The implications and utilization of soluble factors in cancer treatment is a topic that in recent years has begun to gain traction as an important area for investigation.

Intercellular interactions are also critical to establish and maintain the tumor immunosuppressive barrier, by excluding or incapacitating host immune cells. For example, expression of pro-apoptotic molecules, such as FasL can target infiltrating effector T cells in the tumor environment, while sparing apoptosis resistant tumor cells, CAFs, and cancer-associated endothelial cells. For more information on immune cells within the TME, please see the next section on metabolism, vascular invasion, and immune cells within the TME.

## Metabolism, Vascular Invasion, and Immune Cells Within the TME

Formation of blood vessels is an absolute requirement for tumor growth, survival, and progression. Without access to oxygen and nutrients supplied by the blood, tumor growth is restricted to an ~1–3 mm diameter mass *ex vivo* and ~100–500 microns *in vivo* (76–78). It stands to reason that the aspects of the TME reviewed might precede angiogenesis, lymphangiogenesis, and immune invasion due to the size of the tumor where these processes occur. Tumor neovascularization is a complex and multifaceted process driven by tissue hypoxia, defined as tissue with oxygen concentrations below 10 mmHg, which is a common feature of solid tumors (79, 80). Below this threshold, cells upregulate a host of adaptive proteins; a response mostly driven by the heterodimeric transcription factor, hypoxia-inducible factor (HIF-1) (81). In normoxic conditions, prolyl hydroxylases (which have oxygen dependent enzymatic activity), hydroxylate proline residues in the oxygen degradation domain of HIF-1α. The von Hippel-Lindau (VHL) complex is then able to recognize HIF-1α for subsequent proteasomal degradation (81). Under hypoxic conditions, VHL itself undergoes proteasomal degradation, leading to stabilization of HIF-1α and subsequent binding to its constitutively regulated partner, HIF-1β (82). Once this occurs, the HIF-1α/HIF-1β heterodimer enters the nucleus and binds to hypoxia-regulated-elements (HREs) of hundreds of genes (83). Binding targets of HIF-1 are in part controlled by epigenetic changes that promote active chromatin states at HIF binding sites (84). The impacts of HIF-1 binding are numerous, from reducing oxygen consumption to increasing angiogenesis through regulation of vascular endothelial growth factor (VEGF), the angiopoietin-1 regulated tyrosine kinase receptor TIE2 (also known as TEK), and angiopoietin, among others (83, 85).

**Figure 3:**
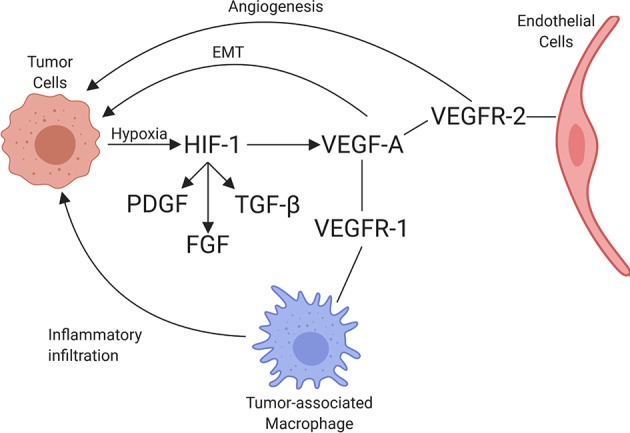
Schematic of the interaction between tumor cells and endothelial cells, including the associated recruitment of inflammatory cells by endothelium. Through hypoxia, HIF-1 is upregulated, increasing PDGF, FGF, TGFβ, and VEGF-A expression. VEGF-A binds to its receptors, VEGFR-1 and VEGFR-2, leading to angiogenesis (through VEGFR-2 signaling), immune cell invasion (through VEGFR-1 signaling), and promotion of epithelial-mesenchymal transition (EMT) in tumor cells (created with Biorender.com).

VEGF is a potent growth factor influencing vascular permeability and angiogenesis (86). VEGF-A is one of the best-characterized forces in the development of new vessels and binds to VEGF receptors-1 and -2 (VEGFR-1 and VEGFR-2). VEGF-A can be secreted along with other pro-angiogenic factors by numerous cell types, including adipocytes, within the TME (87). VEGFR-1 and VEGFR-2 are both receptor tyrosine kinases that contain a split tyrosine-kinase domain, although they function differently within the TME (88). VEGFR-2 is upregulated in endothelial cells of newly forming blood vessels within tumors and is commonly implicated in neovascularization. A recent study demonstrated that the α4β1-integrin is capable of VEGFR2 binding and activation, presenting a novel potential target for therapy (89). Alternatively, VEGFR-1 has relatively weak pro-angiogenic properties and can recruit and activate tumor-associated macrophages (TAMs) and myeloid cells, promoting tumor cell metastasis and proliferation [Figure 3; (90)]. Little is known about the dynamic balance between VEGFR-2 and VEGFR-1 in tumors of dogs, but there is one report suggesting that heritable traits or the breed background might influence the expression and function of these receptors in vascular sarcomas (91). A second major regulator of angiogenesis is Tie-2. In the presence of active Tie-2 signaling, the vasculature remains in a mature state surrounded by pericytes (92). Angiopoietin 2 (ANGPT2), an angiopoietin 1 competitive antagonist and HIF-1 target gene, binds to endothelial cells, preventing Tie-2 signaling. This causes the vasculature to become less mature with fewer pericytes (93). This microvasculature is then primed for maximum response to VEGF.

Cyclo-oxygenase 2 (Cox-2), which is involved in the formation of some types of prostaglandin production, has been shown to increase expression of VEGF-A mRNA in tumors, thus having pro-angiogenic properties (94). The mechanism by which Cox-2 increases VEGF-A expression in tumors likely involves p38 mitogen activated protein kinase (MAPK) and Janus kinase (JNK) pathways. These pathways are integral in the transcriptional and post-transcriptional regulation of VEGF-A (95, 96). Elevated Cox-2 levels have been reported in canine and human prostatic carcinoma, transitional cell carcinoma, and squamous cell carcinoma, among others [Table 1; (97–102)]. As such, anti-Cox-2 therapies, including non-steroidal anti-inflammatory drugs (NSAIDs) which inhibit the production of Cox-2, have been the subject of anti-cancer initiatives. In dogs with urothelial cell carcinoma, treatment with the NSAIDs piroxicam and deracoxib have shown promising clinical results, including decreasing tumor volume and increasing apoptosis of neoplastic cells (103, 104). Similarly, NSAIDs have been used for their anti-tumor effects in humans, such as a chemopreventative agent for colorectal cancer and certain subtypes of breast cancer (105, 106). Another promising anti-tumor therapy that leverages increased Cox-2 expression in tumors utilizes conditionally replicative oncolytic adenoviruses. To overcome the traditionally poor infectivity of these viruses, an oncolytic adenovirus with Cox-2 promoter-based targeting control mechanisms was designed. This viral therapy is specific to Cox-2 positive cells with the potential to specifically target a variety of Cox-2 positive tumors, thereby increasing efficacy and safety of this potential therapy (107, 108).

The clinical implications of HIF-1 and VEGF expression and regulation have been the subjects of recent investigation. Moeller et al. (109) were the first to demonstrate that radiotherapy upregulates HIF-1 protein levels, even at a time when the tumor is re-oxygenated. The mechanism for this effect was shown to be related to two factors: (1) release of HIF-1 mediated transcripts of HIF-1 stored in stress granules during hypoxia, and (2) an increase in oxidative stress after radiotherapy, preventing the activity of prolyl hydroxylases to prime HIF-1α for degradation. As a follow on, Li et al. (110) demonstrated that infiltration of macrophages into irradiated tumors stabilized HIF-1 via a nitric oxide mediated mechanism. Other cytotoxic treatments have been shown to increase HIF-1α levels via mechanisms involving oxidative stress. Doxorubicin can upregulate HIF-1α levels in aerobic tumor cells by stimulating inducible nitric oxide synthase (iNOS) activity (111). Hyperthermia increases the activity of NADPH-oxidase in tumor cells, thereby stabilizing HIF-1α. There are multiple potential consequences of chronic HIF-1 transcriptional upregulation, but of central importance is the upregulation of VEGF. As part of a clinical trial conducted in dogs with soft tissue sarcomas, treated with fractionated hyperthermia and radiotherapy, Chi et al. (112) examined the hypothesis that there would be an increase in HIF-1 mediated transcripts and associated physiologic modification early in the course of treatment. Tumor tissues were removed prior to, and 24 h after, radiotherapy and the first hyperthermia treatment. Tissues were examined for changes in gene expression and, concomitantly, the apparent diffusion constant of water of these tumors was measured using magnetic resonance imaging (ADC-MRI; a biomarker of hyperpermeability). Unsupervised gene expression analysis showed two main groupings, distinguished by whether ADC-MRI increased (indicating increased water content) or remained unchanged. Among several HIF-1 regulated genes observed in the subgroup that showed increased ADC, VEGF upregulation was one of the most predominant (113). Thus, this canine clinical study supported pre-clinical results; that the combination of hyperthermia and radiotherapy increases HIF-1 transcriptional activity. The fact that ADC only increased in a fraction of tumors, suggests that ADC may be a viable biomarker for understanding how the physiologic microenvironment responds to cytotoxic therapy. An important future direction of these observations includes ascertaining whether changes in ADC are associated with treatment outcome.

Hypoxia affects innate and adaptive immune function in multiple and complex ways (114). Macrophage response to hypoxia is multifaceted and relies on the presence and concentration of cytokines and other immune cells. TAMs, which are believed to arise from the resident macrophage pool, have been categorized as “M0” (uncommitted), “M1” (pro-immune), and “M2” (pro-angiogenic and immunosuppressive) (115–117). However, both resident and recruited macrophages are remarkably plastic and can revert among these phenotypes, with all three co-existing in different stages of tumor development and progression. M2 TAMs tend to accumulate in hypoxic regions due to hypoxia-mediated chemokine expression by both tumor and stromal cells (116, 118). The presence of macrophages in hypoxic regions promotes immunosuppressive functions, including release of immunosuppressive cytokines such as TGFβ, recruitment of regulatory T cells (Tregs), and binding of programmed death-1 (PD-1) receptor on cytotoxic T-cells by the HIF-1 target, programmed death ligand-1 (PD-L1) (118, 119). Additionally, hypoxia inhibits the adaptive immune system by downregulating T-cell motility and upregulating the HIF-1 targets SDF-1 and its ligand CXCR4, thereby stimulating intratumoral recruitment of immunosuppressive MDSCs (120–122). Hypoxia disturbs the balance between effector T cells and Tregs, tipping the balance toward the latter (123).

In the absence of oxygen, cells are obligated to use glycolysis to produce ATP. Reprogramming energy metabolism is regarded as a hallmark of cancer, as described by Hananhan and Weinberg (124). The “Warburg Effect,” the unique process of tumor cells utilizing aerobic glycolysis, was first described by Warburg (125). Lactate is the product of both aerobic and anaerobic glycolysis (126). The relative predominance of hypoxia in tumors, therefore, is a major contributor to the production of lactate. In addition, the relative inefficiency in solute transport by tumor vasculature leads to accumulation of lactate to substantially elevated levels. Concentrations of lactate can range from normal levels of 1–2 mM to as high as 15–40 mM in both pre-clinical and human clinical samples (127, 128).

Lactic acid is a major component that fuels metabolic symbiosis between the aerobic and hypoxic tumor compartments (129). Lactate produced by hypoxic tumor cells is transported by passive monocarboxylic acid transporters (MCTs), which enable lactate to be excreted by cells that produce it and to be taken up by aerobic cells where in high concentrations, is back converted to pyruvate, where it enters the tricarboxylic acid (TCA) cycle to produce alanine and glutamate (126, 130). The affinity of aerobic tumor cells for lactate is 10 times higher than glucose, indicating that aerobic tumor cells preferentially use lactate (130). If the ability of aerobic tumor cells to use lactate is blocked, the cells will switch to utilizing glucose, thereby depleting local glucose concentrations. Excess glucose present in aerobic tumor regions can diffuse to hypoxic regions, where the glucose is catabolized to lactate (129). Since hypoxic tumor cells are reliant on glucose, even though some can use glutamine in its stead, this can lead to death of the cell (129, 131).

Utilization of lactate has also been described in tumor-associated fibroblasts, which have low expression of CAV-1, an inhibitor of myofibroblast differentiation (132). An informative study showed that tumor-associated myofibroblasts could use aerobic glycolysis to produce lactate. Lactate was then used by aerobic tumor cells to fuel the TCA cycle through its conversion to pyruvate. The authors termed this symbiosis the “Reverse Warburg Effect” because the myofibroblasts were responsible for aerobic glycolysis instead of the tumor cells (132). Lactate can also stimulate the stabilization of HIF-1α in aerobic tumor and endothelial cells (133). Like aerobic tumor cells, endothelial cells uptake lactate (133). The conversion of lactate to pyruvate interferes with the activity of the prolyl hydroxylases responsible for HIF-1α degradation. In the presence of elevated pyruvate, HIF-1α levels, and consequently VEGF levels increase, which promotes angiogenesis. The negative influence of lactate on cancer prognosis in humans is most likely attributed to downstream stabilization of HIF-1α in tumor and stromal cells (134). Lora-Michiels et al. (135) demonstrated that in 39 dogs with soft tissue sarcoma, those with relatively low pH tumors were associated with shorter progression free interval and overall survival than dogs with higher tumor pH. Extracellular pH, which is simpler to measure, can be used as a surrogate of lactate, since transport of lactate across a cell membrane via the MCT transporters includes a hydrogen ion (135). High lactate levels and associated extracellular acidosis also contribute to immune suppression (136).

Once tumor-associated blood vessels are formed, they are structurally and epigenetically abnormal, which facilitates metastatic spread. These vessels tend to be irregularly dilated and tortuous with increased permeability, decreased pericyte numbers, and abnormal deposition of collagen type IV in the basement membrane. Endothelial cell adhesion molecules, such as selectins and integrins are required for leukocyte transmigration into tissues (137, 138). It has been reported that these adhesion molecules are often absent in tumor microvessels, thereby reducing the ability of immune cells to gain access into tumors (137, 139). The downregulation of adhesion molecules is regulated by VEGF (139) and can be reversed by blocking VEGF or by altering IL-6 trans-signaling (138). Thus, the first line of defense that tumors use to inhibit immune surveillance is the blockade of transmigration of immune cells. Furthermore, there is substantial signaling between endothelial cells and tumor cells, especially CSCs, which have a tendency to seek out or create vascular niches (140). Several signaling pathways, including Sonic Hedgehog, and Notch, to name a few, emanate from endothelial cells and promote acquisition of CSC properties and proliferation within vascular niches (141). In the tumor microenvironment, it is likely that the balance between these pro and anti-inflammatory mediators dictates the extent of leukocyte-endothelial cell interactions that occur naturally and in response to therapy. Modulation of these interactions is likely essential for optimization of immunotherapy.

Like neoangiogenesis, lymphangiogenesis can act as an important gateway to tumor metastasis. The density of lymphatic vessels within a tumor has been correlated with tumor growth and metastasis in both dogs and humans [Table 1; (142–145)]. Mechanistic control of lymphangiogenesis is complex, involving a multitude of factors including many of the same factors described in tumor-associated neovascularization. Two of the major mechanisms controlling lymphangiogenesis are well-described. One is dependent on VEGF-C and VEGF-D produced by both tumor cells and TAMs, which bind VEGFR-3 on lymphatic endothelial cells (LECs) (146). The other is the SRY-related HMG-box (SOX18) pathway through prospero homeobox-1 activation (147, 148). Lymphangiogenic factors not only increase the number of lymphatic vessels within solid tumors, but also are capable of enlarging the diameter of the lymphatic vessels, increasing tumor cell metastasis to local lymph nodes (149). Furthermore, VEGF-C secreted by tumor cells can promote lymphangiogenesis within draining lymph nodes, increasing the number and diameter of lymphatic vessels thereby increasing the overall metastatic potential of the tumor (150). Although little is known about tumor cell entry into lymphatic vessels, multiple studies have demonstrated that cancer cells can express CC-chemokine receptor 7 (CCR7), which lymphocytes use to home to lymph nodes via CCL21 binding, in a sense, hijacking the lymphatic system to gain entry to lymphatic vessels and lymph nodes (151). LECs have additionally been implicated in immunomodulation within the TME, including multifaceted mechanisms to promote immune evasion. These include local deletional tolerance of CD8+ T cells, inhibition of dendritic cell maturation leading to decreased effector T cell activity, and tumor antigen trapping and retainment to archive for antigen-presenting cells (152–154).

In their landmark update of the Hallmarks of Cancer in 2011 (124), Hanahan and Weinberg called tumor-promoting inflammation an enabling characteristic of cancer. Inflammation is critical for the formation and maintenance of the tumor niche. It persists throughout the existence of the tumor, through therapy, remission, stabilization of disease, and relapse, and it is foundational in creating the metastatic niche. In its steady state, inflammation in the TME can promote or inhibit the capacity of innate and adaptive immune cells to infiltrate the tumor stroma and eliminate the tumor cells. However, the inflammatory TME is highly dynamic (155), characterized by a recurring cycle that established an evolutionary arms race at microscopic scale. Whether the balance tips toward immunosuppression or toward productive anti-tumor immunity is a critical determinant in the ultimate rate of tumor progression and patient outcomes.

Thousands of studies have examined the composition of the TME in humans and animals. Most studies focused on one or a few features in isolation, such as infiltration by immunosuppressive elements like Tregs or by tumoricidal NK cells or cytolytic T cells. For example, increased CD4+:CD8+ T cell ratios were correlated with decreased survival in dogs with mammary carcinomas (156), and enrichment of Foxp3+ regulatory T cells within tumors was associated with tumor progression in mammary and testicular cancers (157, 158). As another example, the immunomodulatory properties of MSCs follow licensing by inflammatory cytokines such as interferon-γ (IFNγ) and TNFα (159, 160). Licensed MSCs are resistant to apoptosis, and thus impervious to immune attack. In both syngeneic and xenograft models, MSCs reorganize the TME, excluding T cells, macrophages, and other host effector cells, tilting the balance away from tumor host control and toward tumor progression (161, 162). MSCs are also able to inhibit T cell proliferation and inhibit natural killer (NK) cell function through soluble factors, and cell-cell communication (163–165). Paradoxically, these cells can inhibit TNFα and IFNγ which are initially necessary for licensing or “tumor-mediated education,” while also increasing IL-10, an immunosuppressive cytokine (164). In dogs, MSCs induced from skin fibroblasts have shown similar immunomodulatory effects to naturally sourced MSCs (166).

Recent advances in next generation sequencing and bioinformatics, as well as the availability of high-quality samples that comprise The Cancer Genome Atlas (TCGA), made it possible to divide the immune landscape of human tumors into six distinct steady states or subtypes (167). Thorsson et al. based these subtypes on their respective transcriptional programs (Table 2), which in turn allowed them to establish the predicted cellular composition for each tumor (167). While these subtypes are probably not static, their dominance at any given type in any given tumor is prognostically significant. It should be noted that there was extensive overlap among genes, and therefore among predicted cell types comprising these subtypes, underscoring the futility of trying to understand the relationship between cancer and the immune system without the benefit of comprehensive information. For this reason, the extensive literature describing unique components of the immune TME will not be further summarized in this review. Emerging technologies such as single cell sequencing (168, 169) and highly multiplexed 3-dimensional optical imaging (170, 171), individually and combined, will bring about the next transformation in the understanding of the immune landscape of cancer.

**Table 2:** Immune subtypes of cancer.

	Mϕ:Lymph ratio^*^	**TH1:Th2 ratio**	**Proliferation**	**Intratumoral heterogeneity**	**Other**
Wound healing	Balanced	Low	High	High	
IFNγ dominant	Lowest	Lowest	High	Highest	Highest M1 and highest CD8 T cells
Inflammatory	Balanced	High	Low	Lowest	Highest Th17
Lymphocyte depleted	High	Minimal Th	Moderate	Moderate	
Immunologically quiet	Highest	Minimal Th	Low	Low	Highest M2
TGFβ dominant	High	Balanced	Moderate	Moderate	Highest TGFβ signature

* *Mϕ:Lymph ratio, macrophage to lymphocyte ratio*.

Recent advances in sequencing technology and bioinformatics (172) are being applied to studies of canine immuno-oncology. Specifically, genome-wide gene expression profiles using RNA-Seq transcriptomic data can be utilized to estimate the abundance of distinct subsets of immune infiltrate in the tumor tissues and to examine the features of the inflammatory response (167, 173, 174). Scott et al. (175) showed that, even though osteosarcomas are immunologically “cold” (barren) tumors, RNA sequencing was sufficiently sensitive to detect transcripts (Table 1). This points to the presence of immune cellular infiltrates that allow stratification of spontaneous osteosarcomas of humans and dogs into immunologically “hot” and “cold” tumors. The transcriptional programs associated with immune cells were remarkably well-conserved across tumors from both species and did not show specificity regarding cell type or upregulation of specific molecules, such as those associated with immune checkpoints. While the increased presence of immune transcripts in tumors was associated with significantly better prognosis in human patients, such relationship was absent in dogs (Table 1). This observation is especially paradoxical when considering the reproducible success of experimental immunotherapies in canine osteosarcoma models (176), and even though the basis for it is not yet understood, it raises an important cautionary note in the application of canine osteosarcoma as a model for immunotherapy of human osteosarcoma.

Gorden et al. (177) showed that spontaneous canine hemangiosarcomas can be classified into three distinct molecular subtypes. Preliminary data suggest that these tumors in virtually all dogs that achieved durable remissions after conventional therapy show enrichment of immune gene signatures. Other groups have reported the immune characteristics of canine gliomas (178) and canine malignant melanoma (179), both showing similar patterns of immune infiltration to those reported for bone and vascular sarcomas.

It is widely accepted that macrophages play a major role in molding the TME, making them attractive targets for tumor therapy. Myeloid antigen presenting cells (APCs), and especially dendritic cells, control the initial steps in the cancer-immunity cycle, engulfing tumor cells and tumor debris and presenting it to T cells in the draining lymph nodes (155). Since tumors are derived from “self,” immune recognition can be compromised, and this process can lead to tolerance (155, 180). Immune recognition and activation, however, is aided by genomic instability. Tumors show a direct relationship between mutational burden and immune infiltration, and this relationship extends to the observed response to immunotherapies (167, 181, 182). After immune recognition, T cells must traffic to the tumor, extravasate and infiltrate the tumor stroma, recognize the cancer cells, and selectively kill them (155). Each of these steps creates opportunities for the tumor to inhibit or evade the immune response—and concomitantly, a potential step for therapeutic intervention.

Immune recognition of the tumor intensifies the selective pressures that drive tumor evolution through the process of immunoediting (183). T cells can potentially eliminate the majority of cells in a tumor that display particular mutations. The T cell receptor repertoire in tumors is becoming increasingly well-understood, following conventional roles of clonal evolution. In virally induced tumors, such as those associated with Epstein Barr virus, narrowing of the repertoire through strong selection for foreign viral epitopes is associated with worse prognosis (184). This observation extends to tumors without viral etiologies, where tumor epitopes promote selection of a narrow diversity of clones. Greater clonal heterogeneity is associated with a more favorable prognosis in multiple tumor types (185–187). Lymphocyte clonal diversity and the potential to modulate it therapeutically in canine osteosarcoma has been examined. Preliminary results document feasibility and show variation in the diversity of the T cell repertoire across different tumors (188). However, the influence of clonal diversity on outcomes for dogs with cancer remains to be determined.

The elimination phase of immunoediting gives way to an equilibrium phase where the immune system appears to control the tumor. However, editing is not static, and the process eventually favors outgrowth of resistant tumor cells that “edit” the epitopes recognized by the immune system. Editing can occur through downregulation of major histocompatibility complex (MHC) proteins, upregulation of proteins that resist T cell activation and killing, acquisition of the ability to kill activated T cells or resist T-cell induced apoptosis, or alteration of target epitopes by further mutation or epigenetic regulation (183). The host is able to respond to these immune evasive mechanisms, for example by deploying NK cells that recognize tumor (and other) cells that downregulate MHC (180, 189). However, most cases progress to the third phase of escape (183). The immune system has evolved over millions of years to defend hosts against lethal pathogens, with the function of cancer immunosurveillance probably arising as a secondary benefit (190). Cancer is rare before reproductive age and even into young adulthood, and so robust anti-tumor immunity is unlikely to create sufficiently large selective pressure to favor individual survival.

Some common steps in the evolution of the immune microenvironment of cancer has led to the development of highly effective immunotherapies. Immune checkpoint blockade using antibodies that interfere with binding of cytotoxic T-lymphocyte-associated protein 4 (CTLA-4) to CD80 and CD86, or with binding of PD-1 to PD-L1 and PD-L2, are the first therapies directed against the TME that have been effective at achieving meaningful cancer control. Durable remissions in as many as 50% of patients with advanced cutaneous melanoma, various types of tobacco-related malignancies, gastrointestinal tumors, and certain blood cancers have been achieved via these therapies (182, 191–193). The best responses cluster in tumors (or tumor types) with high mutational burden and robust immune infiltration (182). Multitudes of other immune-enhancing therapies that can modulate the TME, and especially that can shift the inflammatory response toward T helper-1 (Th1) programs are under development, alone or in combination with immune checkpoint blockade. These include Toll-like receptor (TLR) agonists, oncolytic viruses, VEGF inhibitors that promote blood vessel normalization and improve T cell trafficking to tumors, among others (194). Indeed, the first chimeric antigen receptor redirected T cells (CAR T cells) directed against mesothelin, a protein expressed exclusively in the TME, have completed early phase clinical trials (ClinicalTrials.gov Identifier: NCT01583686).

Spontaneous canine cancers provide a rich resource to understand both conserved and species-specific mechanisms that create and maintain the tumor immune landscape. Dogs can teach us much about therapeutic immune system manipulation in the context of cancer, including the potential to alter the TME to enhance immune responses. There are numerous published studies on the subject, including using pharmacologic intratumoral delivery of vectors encoding FasL (195), a variety of tumor vaccine approaches that activate molecular pattern receptors (196–198), applications of CAR-T cell immunotherapies (199), and others (200, 201). That being said, these data must be interpreted with due caution. It should be recognized that the canine and human immune systems are separated by millions of years of evolution and were adapted to distinct pathogens in distinct environments until both species collided into shared social structures about 20,000 years ago (202), that became more intimate over the past three to five decades (203). The timeline of the canine-human relationship is rather short, and the strong influence of artificial selection will inevitably diminish the role of immunosurveillance in adaptive evolution for both species.

## Involvement of the TME in Tumor Invasion and Metastasis

**Figure 4:**
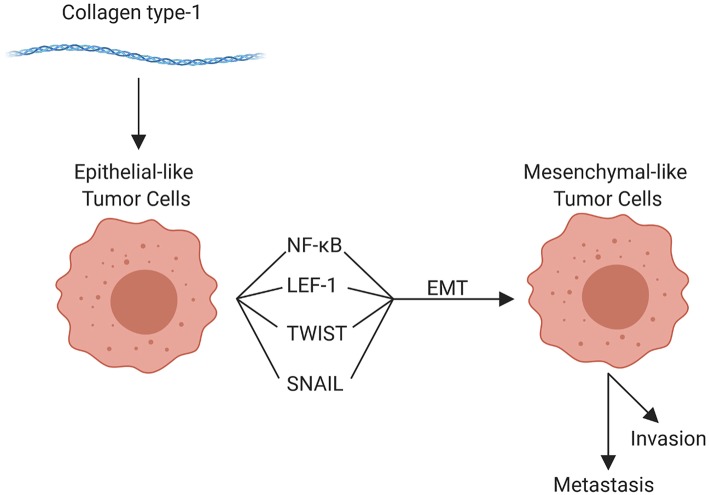
Role of collagen type 1 in the tumor microenvironment and in epithelial-mesenchymal transition (EMT). Collagen type 1 leads to the upregulation of numerous factors that promote EMT, promoting invasion, and metastasis (created with Biorender.com).

The components of the TME work in concert through epigenetic and functional means to promote tumor cell invasion and metastasis. Although metastasis can occur at any point in space and time during the course of tumor evolution, the cellular, structural, and molecular components of the TME are able to enhance numerous pro-tumorigenic activities, which in turn facilitate invasion and enhance the metastatic potential of tumor cells. The organization of the primary tumor niche requires significant alterations to the normal extracellular environment. Molecular interactions are highly specific to different tumors and can vary substantially even within tumor types; but the general features include “loosening” of stable cell-cell adhesion, loss of cell polarity, and reorganization of the cytoskeleton, as well as stiffening of the extracellular matrix, which enhances motility, facilitates invasion, and enables the metastatic phenotype (59, 71, 73). Collectively, these alterations are linked to the “epithelial to mesenchymal transition” (EMT). EMT is characterized by genetic and epigenetic changes that alter expression of genes encoding cadherins, occludins, claudins, integrins, and a multitude of other adhesion and cell surface proteins, as well as cytokines, extracellular proteases, and many others. Reduced expression of epithelial (E) cadherin (CDH1) with a concomitant increase in neuronal (N) cadherin (CDH2) is among the most well-studied features of EMT (12, 15, 64). Loss of E-cadherin, which is widely conserved in epithelial tumors across species, is an indicator of more aggressive behavior and poor prognosis for a multitude of human as well as canine (204, 205) tumors. Tumor cells themselves can enhance EMT potential; for example VEGF-A produced by tumor cells, in contrast to VEGF-A produced by the TME, has been shown to promote EMT (206). Regardless, the influence of the TME on EMT should not be overlooked. For example, collagen type I in the adjacent ECM has been implicated in promoting EMT through numerous mechanisms, including upregulation of NFκB, Snail, and lymphoid enhancer-binding factor-1 (LEF-1) in tumor cells. These factors promote a mesenchymal phenotype with subsequent cell migration [Figure 4; (207)]. Other pro-EMT transcripts, such as TWIST1, are expressed in higher concentration in tumor cells adjacent to collagen dense stroma (208). Additionally, intercellular interactions establish a pro-inflammatory environment where autocrine and paracrine loops, such as those mediated by interactions between colony-stimulating factor-1 (CSF-1) and its receptor in CSCs and TAMs, support the EMT transcriptional programs (209).

Changes in cell-to-cell contacts in sarcomas have been less well-studied, almost certainly due to the rarity of these tumors in humans. The concept of EMT in sarcomas presents a paradox: some, but not all sarcomas exhibit aggressive, rapidly metastatic phenotypes, and cells in these tumors undergo phenotypic changes that resemble EMT. Yet, all sarcoma cells possess a mesenchymal phenotype. This has given rise to a more nuanced vision of EMT, and the reverse process called mesenchymal to epithelial transition (MET), where the transcriptional and epigenetic mechanisms that regulate these transitions give rise to metastable phenotypes that are adaptive (12). In other words, cells acquire these phenotypes in response to environmental cues, as well as to natural selection on a microscopic scale.

Emerging evidence suggests that exosomes are critically important mediators that mold the distant or metastatic tumor niche in blood-derived and solid tumors. Exosomes are formed by inward budding of early endosome membranes by the endosomal sorting complex required for the transport (ESCRT) complex. Mature endosomes, also known as multi-vesicular bodies (MVBs) fuse with plasma membranes releasing exosomes vesicles to the extracellular space. Exosomes circulate systemically and can bind to and merge with other cells, creating a mechanism for horizontal transfer of activated oncoproteins, oncogenic DNA, and oncogenic and regulatory microRNAs (210). For example, CAAs and ad-MSCs, a developmentally plastic cell type that can be derived from, or differentiate to adipocytes within the TME, can produce extracellular vesicles (211, 212). To the authors' knowledge, there are no reports characterizing the role of CAAs in dogs; however, extracellular vesicles from human adipocytes have been shown to enhance tumor cell invasiveness by providing substrates for increased fatty acid oxidation in the tumor cells (211). Tumor exosomes carry biologically active molecules; thus, they can reprogram the activity of cells locally, as well as at distant sites, in essence “conditioning” these sites for metastatic tumor growth. Exosomes contribute to the formation of each component of the primary tumor niche, including the metabolic, immune, hypoxic, and infiltrating regions (68, 213). However, their role in metastatic conditioning makes them attractive targets for diagnosis and therapy. Early data in the field of exosome biology and metastasis showed that secreted exosomes could condition regional lymph nodes to create a favorable metastatic niche for melanoma cells (214). These results have been extensively reproduced in multiple experimental tumor systems, extending to other niches such as liver and lungs (213). Strategies to leverage the capacity and specificity of exosomes to home to the metastatic niche are under development as means to improve delivery and activity of drugs that can delay or arrest metastasis. Perhaps most promising is the use of exosomes in early detection and targeted chemoprevention. Canine osteosarcoma was instrumental in the development of an innovative discovery platform to distinguish RNA signatures in serum exosomes originating, respectively from tumor and host cells (215, 216). While this work is still in the early stages, there is potential that these signatures can be used in rationally designed screening programs aimed at detecting changes in the TME in the earliest stages of tumor formation. Novel therapies may be developed that are able to arrest the development of tumors before they form, creating a completely new outlook on cancer prevention.

## Summary

Cancers are complex and dynamic multicellular tissues; multiple distinct events contribute to initiation, promotion, and progression. Ultimately, these events converge into more rigid molecular programs and create recognizable histological tumor phenotypes that are widely conserved across species. Tumor formation is a tightly orchestrated process, molded by selection to support growth and survival of a clonal population of immortalized cells. This review has demonstrated the complexity and intricacies of the TME in the human and mouse, and established, to the best of the author's abilities, the same complexities within the dog. While there is much room for growth in the understanding of the TME in the dog, the current knowledge base in conjunction with the information known about the TME in humans and mice, provides a solid foothold for the development of further basic and clinical endeavors.

## Author Contributions

KL, JK, MD, and JM contributed to the writing of this manuscript. Figures were rendered by KL and AS. Editing was performed by all authors of this paper.

### Conflict of Interest

The authors declare that the research was conducted in the absence of any commercial or financial relationships that could be construed as a potential conflict of interest.
